# Hypoxia signaling in the equine small intestine: Expression and distribution of hypoxia inducible factors during experimental ischemia

**DOI:** 10.3389/fvets.2023.1110019

**Published:** 2023-02-24

**Authors:** Nicole Verhaar, Nicole de Buhr, Maren von Köckritz-Blickwede, Katrin Dümmer, Marion Hewicker-Trautwein, Christiane Pfarrer, Franziska Dengler, Sabine Kästner

**Affiliations:** ^1^Clinic for Horses, University of Veterinary Medicine Hannover, Hannover, Germany; ^2^Department of Biochemistry, University of Veterinary Medicine Hannover, Hannover, Germany; ^3^Research Center for Emerging Infections and Zoonoses, University of Veterinary Medicine Hannover, Hannover, Germany; ^4^Institute of Pathology, University of Veterinary Medicine Hannover, Hannover, Germany; ^5^Institute for Anatomy, University of Veterinary Medicine Hannover, Hannover, Germany; ^6^Institute of Physiology, Pathophysiology and Biophysics, Department of Biomedical Sciences, University of Veterinary Medicine Vienna, Vienna, Austria; ^7^Small Animal Clinic, University of Veterinary Medicine Hannover, Hannover, Germany

**Keywords:** horse, jejunum, mucosa, ischemia, strangulation, hypoxia, HIF, postconditioning

## Abstract

**Introduction:**

Hypoxia inducible factors (HIF) are widely researched in human medicine for their role in different disease processes. The aim of this study was to investigate the expression and distribution of HIF in experimental small intestinal ischemia in the horse.

**Methods:**

In 14 horses under general anesthesia, segmental jejunal ischemia with 90% reduction in blood flow was induced. The horses were randomly divided into two groups of seven horses, one subjected to ischemic postconditioning (IPoC) by delayed reperfusion, and a control group (group C) undergoing undelayed reperfusion. Intestinal samples were taken pre-ischemia, after ischemia and after reperfusion. Following immunohistochemical staining for HIF1α and -2α, the immunoreactivity pattern in the small intestine was evaluated by light microscopy, and the mucosal enterocyte and muscularis staining were semi-quantitatively scored. Additionally, mucosal HIF1α protein levels were determined by an Enzyme Linked Immunosorbent Assay (ELISA), and mRNA levels of HIF1α and its target genes by a two-step real-time Reverse Transcriptase Polymerase Chain Reaction. Statistical comparison was performed between the groups and time points using parametric and non-parametric tests (*p* < 0.05).

**Results:**

All cell types exhibited cytoplasmic and nuclear immunoreactivity for HIF1α. After reperfusion, the cytoplasmic staining of the crypt and villus enterocytes as well as the villus nuclear staining significantly increased, whereas the perinuclear granules in the crypts decreased. The protein levels showed a significant decrease in group C at reperfusion, with lower HIF1α levels in group C compared to group IPoC during ischemia and reperfusion. No other group differences could be detected. In the HIF2α stained slides, mild to moderate cytoplasmic staining yet no nuclear immunoreactivity of the enterocytes was observed, and no significant changes over time were noted.

**Discussion:**

the changes in HIF1α immunoreactivity pattern and expression over time suggest that this transcription factor plays a role in the intestinal response to ischemia in horses. However, the current study could not identify an effect of IPoC on HIF distribution or expression.

## 1. Introduction

Many studies have been dedicated to the treatment of intestinal ischemia reperfusion injury (IRI) in horses, as this disease is associated with a relatively high complication and mortality rate (1). In humans and rodents, hypoxia-inducible factors (HIF) have been identified as important mediators of the response to ischemia, directing many transcriptional responses to hypoxia (2, 3). There are three different α-subunits, HIF1α, HIF2α, and HIF3α, that are constitutively expressed, but oxygen-dependently degraded under normoxic conditions (4). When this degradation is impaired under hypoxic conditions, the α subunit translocates to the nucleus, where it heterodimerizes with the common HIFß subunit to form the active transcription factor (5). Both HIF1α and HIF2α have been the focus of many experimental and clinical studies, because of their role in the modulation and progression of neoplasia, inflammation and ischemia related tissue injury (6–8). Evaluating HIF1α in the intestine, human cell culture and experimental rodent studies have shown that it can modulate epithelial barrier function (9–11) and intestinal inflammation (12, 13). HIF1α also appears to mediate a response to intestinal ischemia reperfusion injury (IRI), with HIF1α expression increasing in ischemic mucosal tissue (14, 15). This may activate protective mechanisms, although it has also been identified as part of a pathogenic inflammatory response (16, 17). In the equine intestine, HIF1α has only been investigated in jejunal tissue oral or aboral to strangulating lesions. One study reported decreased HIF1α expression in manipulated tissue compared to control samples (18), while another found no difference in HIF1α expression (19). Therefore, the significance of HIF1α in the equine intestine and IRI remains unclear.

HIF2α has also been extensively studied in the intestinal tissue of humans and laboratory animals. It was found to be a key regulator in iron absorption (20), and it is overexpressed in chronic inflammatory diseases such as ulcerative colitis and Crohn's disease (21). HIF2α increases under hypoxic *in vitro* conditions in different rodent tissues including intestine (22). However, HIF2α has not been studied in intestinal IRI, and no studies have been published on HIF2α expression or distribution in equine tissues. The same can be said for HIF3α. Moreover, the expression of this HIF subtype has not been consistently identified in the gastrointestinal tract (23, 24).

HIF1α has been the subject of many studies investigating the concept of ischaemic conditioning. Ischemic postconditioning (IPoC) refers to the reocclusion of blood supply following the reduction of a primary ischemic insult. This treatment strategy was shown to ameliorate IRI in different species and tissues (25–27). The signaling role of HIF1α in its mechanism of action is point of discussion, with different authors attributing the protective action of IPoC to either an increase or a decrease in HIF1α expression (26, 28–30). HIF2α has not been investigated in this context.

With many questions surrounding HIF and their mechanism of action, the objective of this study was to investigate the distribution and expression of HIF1α and−2α in experimental small intestinal ischemia in horses. A second objective was to evaluate the role of HIF following the implementation of IPoC in this experimental model. The authors hypothesized that both HIF1α and HIF2α levels would increase during ischemia and reperfusion, and that this response would be more pronounced in the animals subjected to IPoC.

## 2. Materials and methods

### 2.1. Experimental design

The study was reviewed by the Ethics Committee for Animal Experiments of Lower Saxony, Germany, and approved according to §8 of the German Animal Welfare Act (LAVES 33.8-42502-04-18/2856). A power analysis was performed prior to commencing the study using free available software (G^*^Power 3.1.9.2, *Heinrich Heine Universität, Düsseldorf, Germany*). To detect a difference in immunohistochemistry score with an effect size of 1.5, a sample size of 7 horses per treatment group was required, based on a power of 0.8 and alpha of 0.05. Fourteen horses, owned by the university, were randomly assigned to a group subjected to postconditioning (group IPoC, *n* = 7) and an untreated control group (group C, *n* = 7).

### 2.2. Animals

All horses were systemically healthy, and were to be euthanized due to severe orthopedic problems. The horses were stabled at the facilities of the university at least two weeks prior to surgery. The horses had free access to hay and water and were hand walked daily. Group C consisted of five Warmbloods, one Islandic horse and one Thoroughbred, with a mean age of 12.6 ± 8.7 years and mean body weight (BW) of 535 ± 89 kg. Group IPoC consisted of four Warmbloods, one Islandic horse, one Thoroughbred, and one Standardbred, with a mean age of 10.4 ± 8.6 years and BW of 506 ± 96 kg.

### 2.3. Anesthesia and surgical procedure

General anesthesia was induced with 0.1 mg/kg BW diazepam (Ziapam 5 mg/kg, *Ecuphar GmbH, Greifswald, Germany*) and 2.2 mg/kg BW ketamine (Narketan, *Vétoquinol GmbH, Ismaning, Germany*) after premedication with 0.7 mg/kg BW xylazine (Xylavet 20 mg/ml, *CP-Pharma GmbH, Burgdorf, Germany*). Anesthesia was maintained with isoflurane (Isofluran CP, *CP-Pharma GmbH*) in 100% oxygen, and continuous rate infusions with lactated Ringer's solution (Ringer-Laktat EcobagClick, *B. Braun Melsungen AG, Melsungen, Germany*) and dobutamine (Dobutamin-ratiopharm 250 mg, *Ratiopharm GmbH, Ulm, Germany*) were given to effect, to maintain the mean arterial blood pressure between 60 and 80 mmHg. A routine pre-umbilical median laparotomy was performed in dorsal recumbency following aseptic preparation. Segmental small intestinal ischemia was induced in 1.5 m jejunum by occlusion of the mesenteric vessels with umbilical tape. The ligature was tightened under monitoring of intestinal microperfusion with microlightguide spectophotometry and laser Doppler flowmetry (O_2_C, *LEA Medizintechnik GmbH, Giessen, Germany*), and the ligature was tied when the blood flow was reduced by 90% of the pre-ischemic measurement. The ischemia was maintained for 90 min. In group C, the ligature was released without manipulation of the vessels and reperfusion was initiated without delay. In group IPoC, postconditioning was implemented after release of ischemia by clamping the mesenteric vessels for three cycles of 30 s, alternated with 30 s of reperfusion. This was followed by 120 min of reperfusion in both groups. Subsequently, the horses were euthanized with 90 mg/kg BW pentobarbital intravenously (Release 50 mg/mL, *WDT eG, Garbsen, Germany*) without regaining consciousness.

### 2.4. Sample collection and preparation

#### 2.4.1. Immunohistochemistry

Full thickness intestinal samples were taken at the end of the pre-ischemia period (pre-ischemia sample, P), at the end of ischemia (ischemia sample, I), and at the end of reperfusion (reperfusion sample, R). At reperfusion point, an additional sample was taken just proximal to the post-ischemic intestinal segment (proximal sample, PR). One segment of each sample was fixed in a 4% formaldehyde solution for 24–36 h and subsequently embedded in paraffin.

Immunohistochemical staining was performed for HIF1α and HIF2α. In short, the slides were deparaffinized and subsequently the antigen retrieval was done using citrate buffer with a pH of 6.0 at 95°C for 20 min, followed by blocking for unspecific binding with 20 % goat serum. The slides were incubated overnight with 1:500 polyclonal rabbit antibody against HIF1α (HIF-1 alpha Antibody NB100-134 1.0 mg/ml, *Novus Biologicals LLC, Centennial, USA*) or 1:100 monoclonal mouse antibody against HIF2α (Anti-Hypoxia Inducible Factor 2 α Antibody clone 190b, *Sigma Aldrich, Darmstadt, Germany)*. Subsequently, the slides were incubated with secondary antibody (1:200 goat-anti-rabbit or 1:200 goat-anti-mouse, respectively), followed by incubation with the ABC reagent (Vectastain ABC, *Biozol diagnostics Vertrieb GmbH, Eching, Germany*). As negative isotype control, the control slides were incubated with rabbit IgG (IgG from rabbit serum I5006, *Sigma Aldrich*) for HIF1α and with mouse IgG1 (Clone MOPC-21, *BioLegend, San Diego, USA*) for HIF2α instead of the primary antibody. Equine kidney tissue and equine squamous cell carcinoma tissue was used as a positive control. The slides were incubated with 3,3′-diaminobenzidine and counterstained with modified hematoxylin (Delafield Hemalaun).

All slides were scanned to a digital format using a microscopic scanner at 20× magnification (Axio Scan.Z1, *Carl Zeiss GmbH, Oberkochen, Germany*), and subsequently evaluated using the accompanying software (Zen Blue 3.0, *Carl Zeiss GmbH*). In addition to the descriptive evaluation, a semi-quantitative score was developed for comparison between the groups and time-points. The enterocytes in the crypts and the villi were separately graded for staining intensity of both the cytoplasm and the nucleus with the following score for immunoreactivity: grade 0–no staining; 1–weak staining (staining hardly visible); 2–mild staining (light brown); 3–moderate staining (medium brown); 4–intense staining (dark brown; Supplementary file 1). To quantify the difference between the cytoplasmic and nuclear staining within one slide, the nucleus/cytoplasm ratio was calculated. The same score was used for the myocytes of the tunica muscularis. Microscopic photographs were used as color reference, and the evaluation was performed at fixed color settings by one observer, who was blinded for the identity of the slides. Because many sections showed a varying amount of focal cytoplasmic staining close to the nucleus, a separate score was added to quantify the proportion of cells with this perinuclear staining: grade 0–<1%; 1–1 to 25%; 2–26 to 50%; 3–51 to 75%; 4–76 to 100%.

#### 2.4.2. Enzyme linked immunosorbent assay

To quantify the mucosal protein level of HIF1α, an enzyme linked immunosorbent assay (ELISA) was performed. For this purpose, mucosal tissue sections from each sample were snap frozen in liquid nitrogen, and stored at −80°C until further processing. The mucosal tissue was then homogenized in a lysis buffer (NP40 lysis buffer: 150 mM NaCl, 1.0 % NP-40 (Nonidet P40, *Boehringer Mannheim, Mannheim, Germany*, #1332473), 50 mM Tris pH 8.0, 5 mM EDTA, 1× Protease inhibitor mix, *SIGMA-Aldrich, St. Louis, MO, USA*, #P8340) using a high-speed homogenisator (FastPrep-24™ 5G, *MP Biomedicals Germany GmbH, Eschwege, Germany*). Protein content of each individual homogenized sample was assessed by performing a Bradford assay, as described previously (31). A commercial ELISA Kit (Horse hypoxia inducible factor 1, alpha subunit ELISA Kit, *MyBioSource, San Diego, California, United States*) was performed in accordance with the manufacturer's instructions. Only a small alteration in the protocol was made, diluting the HIF protein standard 1:1 in NP40 buffer, to correct for the presences of this buffer in the samples. Optical density was measured using a microplate reader set to 450 nm (Multiscan GO, *Thermo Fisher Scientific GmbH, Dreiech, Germany*). The accompanying software (SkanIt Software 6.0.2 for Microplate Readers RE, ver. 6.0.2.3) was used to plot the concentration curves, with *r* > 0.97 considered acceptable. The measured values were within the detection limit and standard range of the kit. To correct for differences in protein content between the individual samples after homogenisation, the HIF protein level was expressed in pg per mg protein in the sample.

To ensure that the HIF protein levels in the samples were not decreased due to generalized protein degradation in the sample, the housekeeping protein Heat Shock Protein 70 (HSP-70) was also determined in the same samples with a commercial ELISA kit (Horse Heat Shock Protein 70 ELISA Kit, *MyBioSource, San Diego, California, United States*). This was performed in accordance with the manufacturer's instructions using the same small alteration as described above for the HIF ELISA, as well as the same measurement and calculation.

#### 2.4.3. Real-time reverse transcriptase polymerase chain reaction

To investigate a possible upregulation of *HIF1A, GLUT1, EGLN1*, and *EGLN3*, a two-step real-time Reverse Transcriptase Polymerase Chain Reaction (RT-qPCR) was performed. The RNA was extracted from 10 mg of mucosal tissue that had been snap frozen in liquid nitrogen and stored at −80°C until further processing using the ReliaPrep™ RNA Miniprep System (*Promega, Mannheim, Germany*) following the manufacturer's protocol. The RNA concentration and quality were determined with the aid of a spectrophotometer (BioPhotometer, *Eppendorf, Wesseling-Berzdorf, Germany*). In total, 1 μg of high-quality RNA was used for cDNA synthesis using the GoScript^TM^ Reverse Transcriptase Kit (*Promega*) according to the manufacturer's instructions with a MJ Research PTC-200 Peltier Thermal Cycler (*Bio-Rad, Feldkirchen, Germany*). For qPCR, the resulting cDNA was diluted 1:20 and 2 μl were used in a 20 μl reaction volume containing 10 μl of a ready-to-use premix of SYBR Green I dye, dNTPs, stabilizers and enhancers (GoTaq^®^, *Promega*), 112 nM primer mix, and DNase-free water. These mixtures were pipetted in strip tubes (0.1 ml Strips, *LTF Labortechnik, Wasserburg, Germany*) and processed in a Corbett Rotor-Gene 6000 (*Qiagen, Hilden, Germany*) at individually optimal protocols. A no template control with DNase-free water instead of cDNA was applied for each run. qPCR reactions for each sample and gene were run in duplicate to minimize dispensation artifacts. The deviation of Cq of the technical replicates was <0.3. If it was higher, data were discarded, and the run was repeated. The PCR cycles were run using automatic fluorescence emission following each PCR cycle and the amplification specificity was checked after each run by melting curve analysis. The primer sequences and conditions for qPCR are shown in Table 1; the denaturation temperature was always 95°C and the extension was performed at 60°C. The primers were designed with the Primer BLAST tool from the National Center for Biotechnology Information (NCBI, Bethesda, MD, USA) according to known sequences from the basic local alignment search tool (BLAST) in the gene bank database of the NCBI and synthesized by Eurofins MWG (*Ebersberg, Germany*). The amplicons were sequenced again and the product sequences were verified by BLAST. The quantification cycle and amplification efficiency of each amplification curve were determined using the Rotor Gene 6000 Series Software 1.7 (*Corbett/Qiagen, Hilden, Germany*). The amplification efficiency was 100 ± 5% for all genes, except for EGLN3, where it was 88 ± 5%. For analysis of the data, the “relative expression software tool” (REST 2009-RG Mode, *Qiagen*) established by Pfaffl et al. (32) was used to calculate the relative mRNA expression with reference to pre-ischemia, of which the expression was set to 1. The *C*_q_ values set by the software were applied after checking them optically. Normalization of the samples was achieved using the same amounts of RNA and cDNA for processing and by normalizing the data for the target genes with the aid of the geometric mean of the reference genes' hypoxanthine guanine phosphoribosyltransferase (HPRT)1 and ribosomal protein (RPL)4 *C*_q_s. The reference genes have been proven to be stable under the experimental conditions applied in our study. Their stability was tested using the program BestKeeper© (Version 1 by M.W. Pfaffl, *Institute of Physiology, Center of Life and Food Sciences, TUM-Weihenstephan, Germany*) with a power of 1.95 and 2.04 for RPL4 and HPRT1, respectively (*p* < 0.001).

**Table 1:** qPCR primer sequences and conditions.

**Gene name**		**Primer sequence**	**Annealing temp. [°C]**	**Gene bank accession no**.	**Amplicon length [bp]**	**Tm [°C]**
EGLN 1	F	TGGAACAGGTTATGTACGCCA	58	XM_014733482.2	105	79.5
	R	ACCTCCACTTACCTTGGCATC				
EGLN 3	F	TTCATAGCGGATGTGGAGCC	59	XM_003363671.4	101	83.5
	R	AGCGTATCTGGTCGCATAGG				
GLUT1	F	TACGTGGAGCAACTCTGTGG	59	NM_001163971.2	119	84.5
	R	AATCTCATCGAAGGTCCGGC				
HIF1α	F	CCACTCAGGACACGGACTTAG	60	XM_023627857.1	117	83.5
	R	GGGCTTGAAGAACTGCTTTCC				
HPRT1	F	GGGATTTGAATCACGTTTGTGTC	60	XM_023634464.1	94	79.5
	R	CTCCAGATGTTTCTCCAACTCAACC				
RPL4	F	CCAGGCCAAGAATCACAAACTC	60	XM_001497094.4	108	85
	R	TGCTTTCTTCCCTACCACAGG				

### 2.5. Data analysis

An *a priori* power analysis was performed based on expected differences in intestinal immunoreactivity using the program G^*^Power (Version 3.1.9.6; Heinrich Heine Universität, Düsseldorf, Germany). To detect 1 grade difference in histology score between the two treatment groups using a Mann-Whitney test, anticipating 0.6 grade as standard deviation, the sample size was seven horses per group with a total of 14 horses with an alpha of 0.05 and power (1 – beta) of 0.8.

Statistical analysis and graph design were performed using commercial software (Graphpad Prism 9.3, *Graphpad Software Inc., San Diego, California, USA*). For the continuous variables, normal distribution was assessed with the Shapiro Wilks test and by visual inspection of QQ plots of the model residuals. If data were not normally distributed, log-transformation was performed and the transformed data tested for normal distribution. The normal and log-normal distributed data were expressed as mean (±SD). The semiquantitative scores (ordinal) or data that did not show normal or lognormal distribution, were expressed as median (min–max). The equality of variances was tested by visual assessment of the homoscedasticity plots, and by performing Levene's test. Statistical significance was set at *p* < 0.05.

For analysis of the normal and lognormal distributed data, a two-way repeated measures ANOVA was performed for one independent effect (group), and the time points as repeated effect. This was implemented to compare the values between the different time points and groups, with the horses as subject effect. The Geisser-Greenhouse correction was applied for the *p*-values. Multiple pairwise comparisons were performed with a *post-hoc* Tukey test to compare the different time points within the groups, and a *post-hoc* Sidak test for group comparison.

For the log normally distributed qPCR results, the ROUT outlier test was implemented with the maximum desired Discovery Rate (*Q*) set at 1%. After testing for equality of variance, mixed effect model fitted as a two-way repeated measures ANOVA for missing values was performed for one independent effect (group), and the time points as repeated effect. *Post hoc* testing was performed as described above.

For the ordinal and not (log)normally distributed data, distribution free non-parametric models were used for independent (group) and correlated (time points) effects. A Mann–Whitney-*U-*test was executed to compare the results between the different groups at each time point. For comparing the correlated different time points, a Friedman test in combination with the *post hoc* Dunns-test for multiple pairwise comparisons were performed.

## 3. Results

### 3.1. Immunohistochemistry

#### 3.1.1. HIF1α

**Figure 1:**
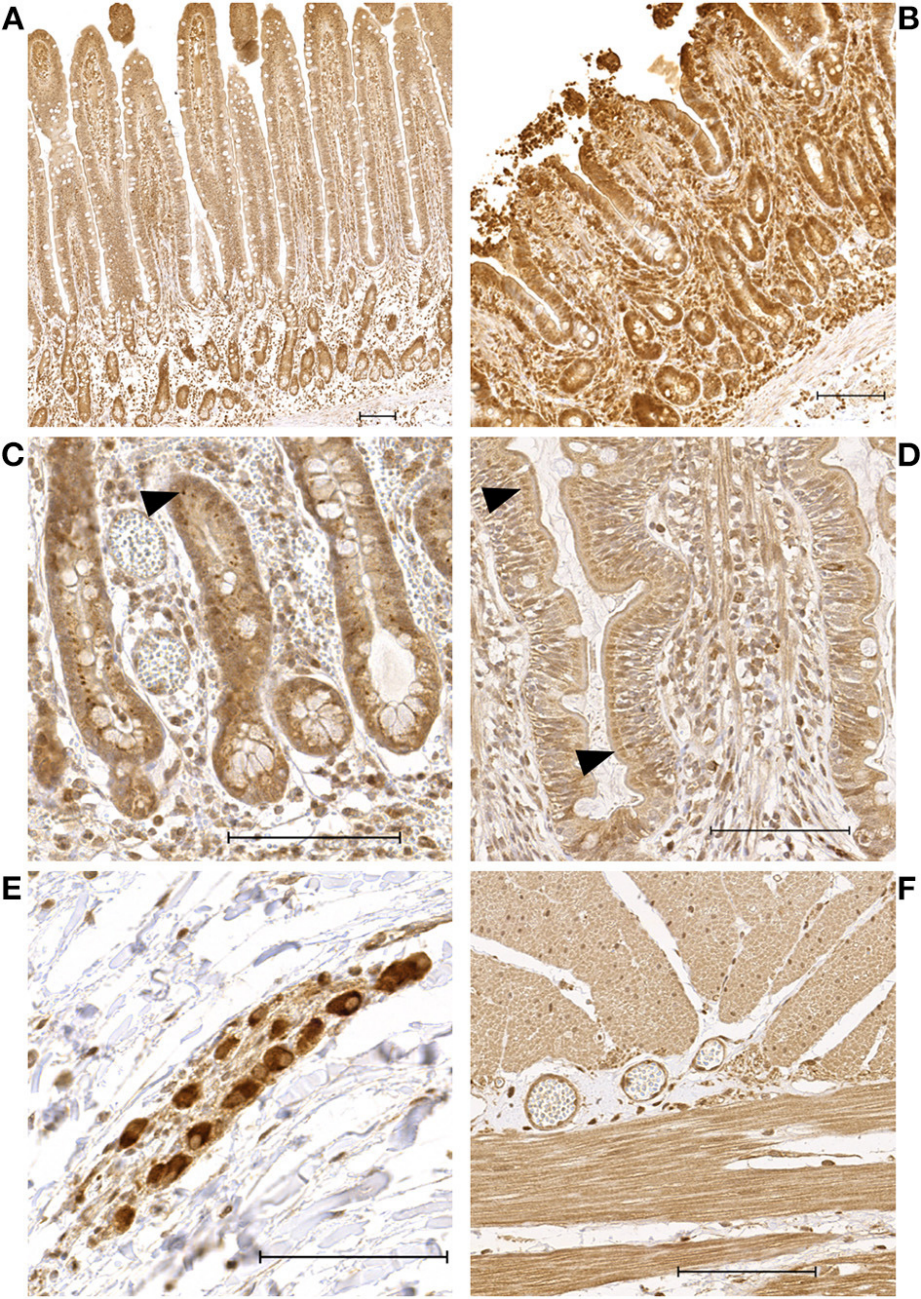
Microscopic images of HIF1α stained sections showing representative examples of the immunoreactivity patterns in the jejunum of horses subjected to experimental ischemia. The scale bar indicates 100 μm. **(A)** Intestinal mucosa of a pre-ischemia sample, demonstrating more intense enterocyte staining in the crypts compared to the villus. **(B)** Intestinal mucosa belonging to the same horse as sample A, taken after reperfusion. **(C)** Crypt enterocytes with intense focal perinuclear staining (arrowheads). **(D)** Villus enterocytes with moderate perinuclear focal staining in a preischemia sample (arrowheads). **(E)** Neurons of the submucosa plexus with intense staining. **(F)** Myocytes in the tunica muscularis with moderate—intense nuclear and moderate myoplasmic immunoreactivity.

The enterocytes exhibited a mild to moderate cytoplasmic and a mild to intense nuclear staining (Figures 1A, B). Inflammatory cells, endothelial cells, and interstitial cells showed consistent intense nuclear staining throughout all time points and in all sections of the intestinal wall. In most slides, the crypt enterocytes showed more intense nuclear and cytoplasmic staining than the villus enterocytes. In some of the P and PR samples with long villi, a more intense staining at the tip of the villus was observed compared to the middle and base section. A varying proportion of crypt enterocytes exhibited a perinuclear focal accumulation of intense staining (Figure 1C). In the villus enterocytes, a similar phenomenon was observed in some of the samples, with moderate to intense focal perinuclear staining, predominantly seen at the base of the villus (Figure 1D). The neurons of the submucosal and myenteric plexus showed moderate to intense nuclear staining (Figure 1E). In the tunica muscularis, the myocytes showed moderate to intense nuclear and mild to moderate cytoplasmic staining (Figure 1F), sometimes with a patchy appearance. The negative control slides did not exhibit any staining.

There were no significant differences in HIF1α staining scores between the horses with and without IPoC. Therefore, the samples of both groups were pooled for comparison between the different time points (Table 2). The reperfusion samples had a significantly higher score for cytoplasmic staining of both the crypts (*p* = 0.047) and the villi (*p* = 0.016) compared to pre-ischemia (Figures 1A, B). The nuclear staining in the villus enterocytes was also higher in the reperfusion sample (*p* = 0.0081). The score for perinuclear granules in the crypt enterocyte cytoplasm was significantly lower after reperfusion compared to pre-ischemia (*p* = 0.0038), as was the nucleus/cytoplasm ratio in the crypts (*p* = 0.031).

**Table 2:** HIF1α immunoreactivity score in the equine jejunum during experimental IRI.

	**Crypt**				**Villus**				**Muscularis**	
	**Cytoplasm**	**Nucleus**	**Nucl./cytopl**.	**Perinuclear**	**Cytoplasm**	**Nucleus**	**Nucl./cytopl**.	**Perinuclear**	**Cytoplasm**	**Nucleus**
Pre-ischemia	2 (2–3)	4 (3–4)	1.8 (1.3–2)	2 (0–3)	2 (1–2)	2 (1–3)	1 (0.5–2)	0 (0–3)	3 (3)	4 (4)
Ischemia	3 (2–4)	4 (3–4)	1.3 (1–2)	1 (0–3)	2 (1–3)	2 (1–3)	1 (0.5–2)	1 (0–3)	3 (2–3)	4 (3–4)
Reperfusion	3 (2–4)^*^	4 (4)	1.3 (1–2)^*^	1 (0–2)^*^	2.5 (2–4)^*^	3 (2–4)^**^	1.2 (0.7–2)	0 (0–2)	2 (2–3)	4 (3–4)
Proximal	2 (2–4)	4 (3–4)	1.5 (1–2)	1 (0–3)	2 (2–3)	2 (1–3)	1 (0.5–1.5)	1 (0–3)	3 (2–3)	4 (0–4)

The table displays the median (minimum–maximum) score. The asterisks indicate a significant difference compared to the pre-ischemia sample of that variable (^*^p < 0.05; ^**^p < 0.01).

#### 3.1.2. HIF2α

**Figure 2:**
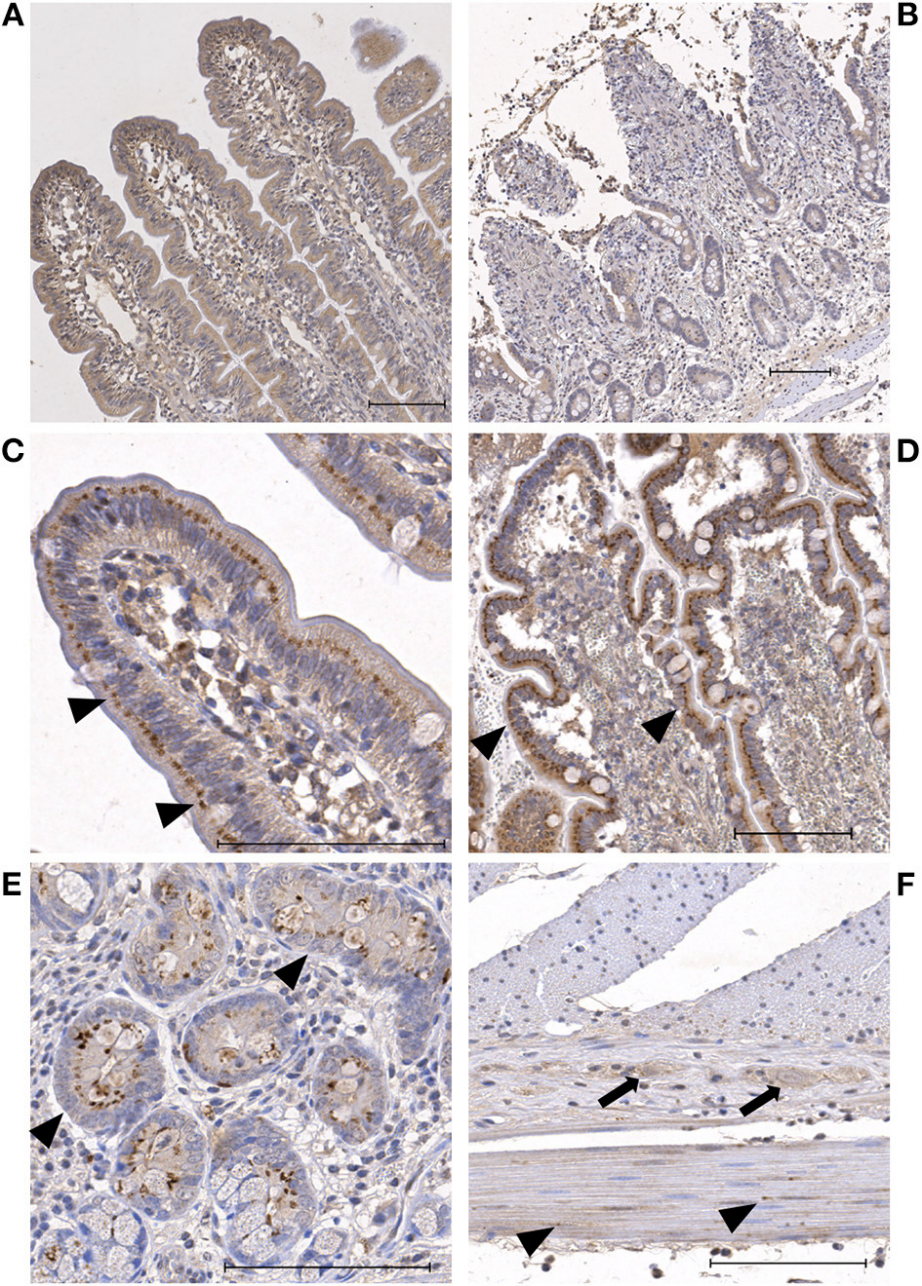
Microscopic images of HIF2α stained sections showing representative examples of the immunoreactivity patterns in the jejunum of horses subjected to experimental ischemia. The bar indicates 100 μm. **(A)** Mucosal villus enterocytes with diffuse mild cytoplasmic staining during pre-ischemia. **(B)** Mucosa of the same horse after ischemia, showing similar mild cytoplasmic staining in the remaining villus enterocytes. **(C)** Focal perinuclear intense staining in villus enterocytes during pre-ischemia (arrowheads). **(D)** Mucosa of the same horse after ischemia, showing similar intense perinuclear staining of the enterocytes (arrowheads). **(E)** Crypt enterocytes with diffuse weak cytoplasmic and focal perinuclear intense staining (arrowheads). **(F)** Neurons in the myenteric plexus with mild staining (arrows) and myocytes in the tunica muscularis with weak diffuse staining and few intensely stained granules (arrowheads).

In the HIF2α stained slides, the enterocytes showed weak to moderate cytoplasmic and no nuclear immunoreactivity (Table 3). In the villus, comparable to the HIF1α stained sections, the enterocytes exhibited two different staining patterns. Most commonly, a diffuse staining of the cytoplasm was seen (Figures 2A, B). In some of the sections, all the villus enterocytes exhibited a moderate—intense focal staining just apical to the nucleus (Figures 2C, D). Subjectively, this appeared to be more intense and crescent shapes at the erosion fronts of some of the villi in the ischemia and reperfusion samples. However, this was not a consistent finding. In the crypts, there were varying amounts of cells with intense perinuclear staining in the enterocytes and goblet cells (Figure 2E). In some of the cells, this focal intense staining formed a crescent shape around the nucleus. The endothelial cells, leucocytes and stromal cells demonstrated weak immunoreactivity in the majority of the slides. The neurons in the submucosal and myenteric plexus were weak to moderately stained (Figure 2F). The myocytes in the tunica muscularis showed a weak to mild cytoplasmic and nuclear immunoreactivity (Figure 2F). Furthermore, varying amounts of moderately to intensely stained granules could be observed, mostly located perinuclear (Figure 2F). In the serosa of the reperfusion and proximal samples, some of the neutrophils demonstrated moderate cytoplasmic and nuclear immunoreactivity.

**Table 3:** HIF2α immunoreactivity score in the equine jejunum during experimental I/R injury.

**Total**	**Crypt**		**Villus**		**Muscularis**	
	**Cytoplasm**	**Perinuclear**	**Cytoplasm**	**Perinuclear**	**Cytoplasm**	**Perinuclear**
Pre-ischemia	1 (0–3)	2 (1–4)	2 (0–3)	1 (0–4)	2 (1–3)	2 (0–3)
Ischemia	1 (1–2)	2.5 (1–4)	2 (1–3)	1 (0–4)	1 (1–3)	1.5 (0–3)
Reperfusion	1 (1–2)	2 (1–4)	2 (1–3)	1 (0–4)	1 (1–2)	1 (0–3)
Proximal	1 (1–2)	2 (1–4)	2 (1–3)	0.5 (0–4)	1 (0–3)	1 (0–3)

The table displays the median (minimum–maximum) score. There were no significant differences between time points.

The mucosal HIF2α immunoreactivity scores can be seen in Table 2, excluding the nuclear score, as this was zero in all samples. There were no significant differences between the groups or time points.

**Figure 3:**
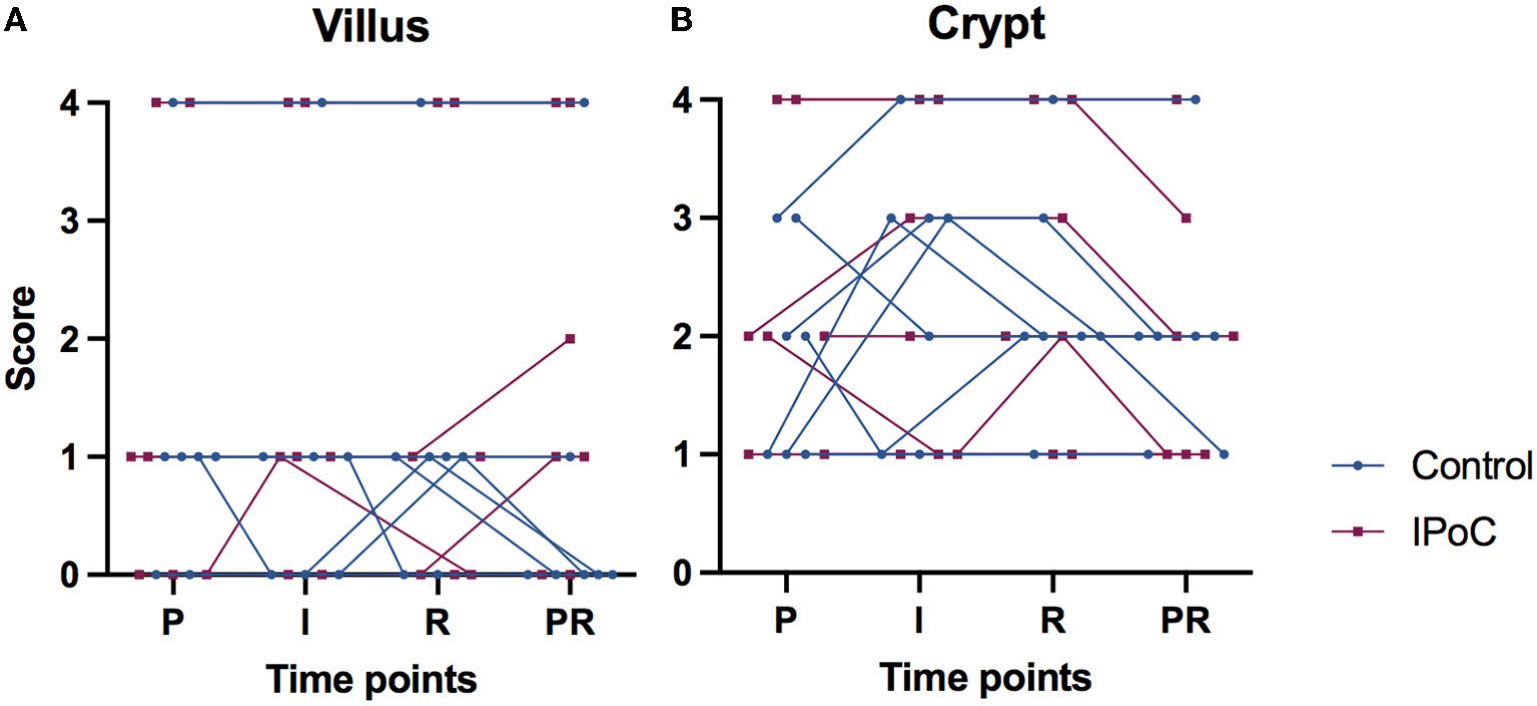
Connected individual value plots of the semi-quantitative score for focal intense perinuclear immunoreactivity with HIF2α in the small intestinal villus enterocytes **(A)** and crypt enterocytes **(B)** of 14 horses subjected to experimental jejunal ischemia. No differences between the groups could be detected. Three individual horses show consistent high scores for perinuclear staining, that do not change over time. P, pre-ischemia; I, ischemia; R, reperfusion; PR, proximal intestinal segment at reperfusion.

Remarkably, there were three horses (one from group C, two from group IPoC) which exhibited a focal intense cytoplasmic staining in all villus enterocytes (Figures 2D, E), which was consistent throughout the samples of different time-points (Figure 3A). On the contrary, the other 11 horses all exhibited scores of 0 or 1 (<25%) for perinuclear focal staining (Figure 3A). Comparably, these three horses had median scores of 4 for the crypt perinuclear focal staining while the other horses had a median of 2 (Figure 3B).

### 3.2. ELISA

**Figure 4:**
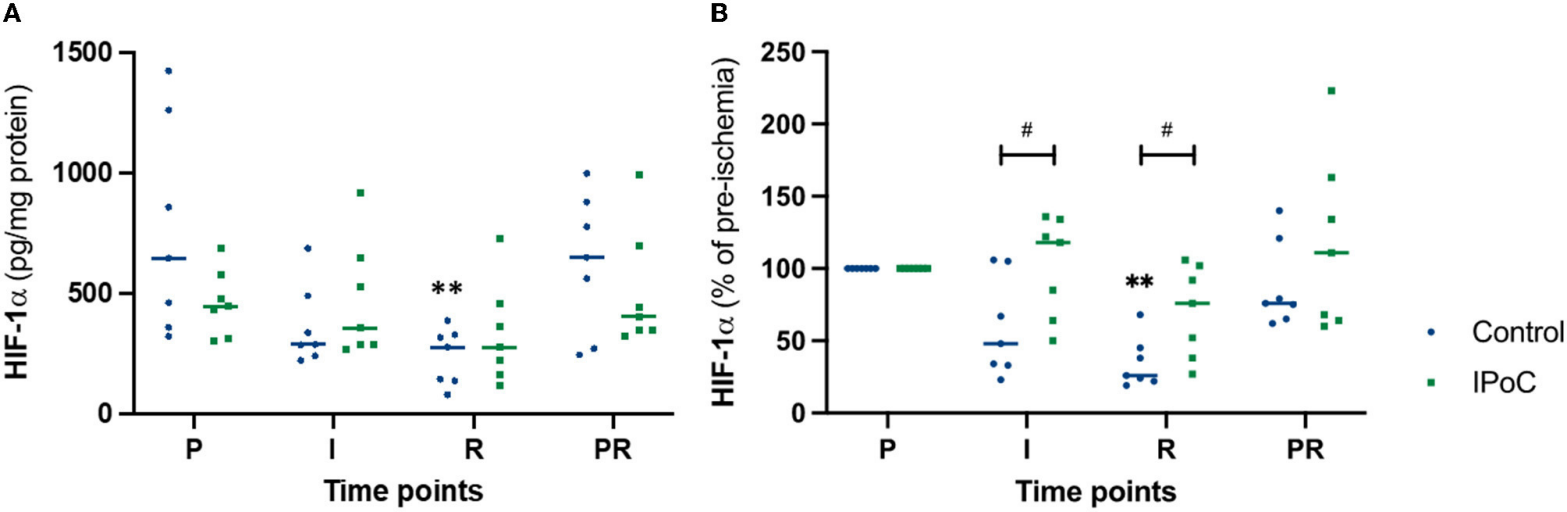
Individual value plots of HIF1α absolute **(A)** and relative **(B)** protein levels in the jejunal mucosa at the following time points: P, pre-ischemia; I, ischemia; R, reperfusion; PR, proximal intestinal segment at reperfusion. In the control group, undelayed reperfusion was initiated following ischemia. In group IPoC, postconditioning was performed by clamping the mesenteric vessels following ischemia. Statistically significant differences of the time point compared to preischemia are marked with an asterisk (**p < 0.01); differences between the groups are indicated with a hashtag (^#^*p* < 0.05).

During pre-ischemia, the HIF1A protein level was 644 (320–1,422) and 445 (301–687) pg/mg protein in group C and group IPoC, respectively (Figure 4A). Looking at the development over time, group C showed a significant decrease at reperfusion compared to pre-ischemia. Due to the varying protein levels in the baseline sample, the relative values compared to pre-ischemia were calculated and used for group comparison (Figure 4B). During ischemia and reperfusion, relative HIF1A levels were lower in group C compared to group IPoC (*p* = 0.038 and *p* = 0.019, respectively).

**Figure 5:**
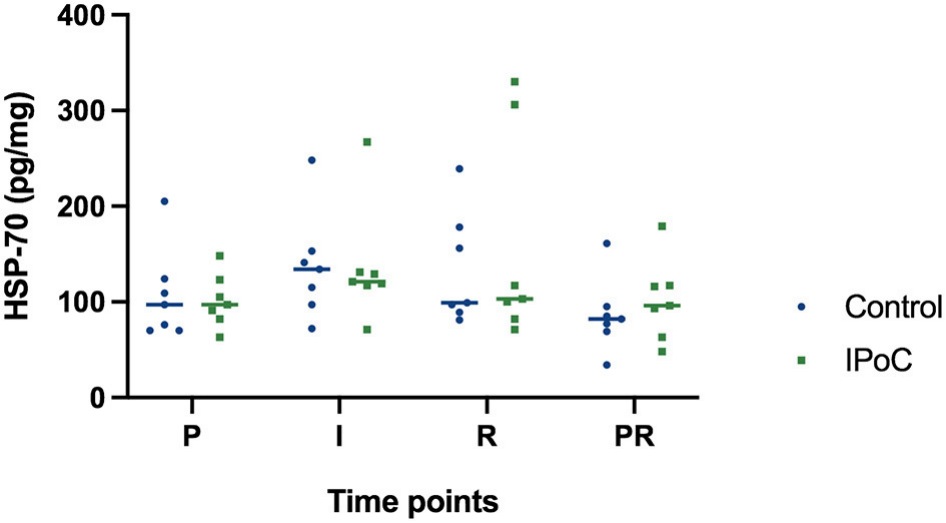
Individual value plot of HSP-70 protein levels in the jejunal mucosa at the following time points: P, pre-ischemia; I, ischemia; R, reperfusion; PR, proximal intestinal segment at reperfusion. In the control group, undelayed reperfusion was initiated following ischemia. In group IPoC, postconditioning was performed by clamping the mesenteric vessels following ischemia. No statistically significant differences could be detected between the time points and groups.

The HSP-70 protein concentration did not show significant differences between the groups or significant changes over time (Figure 5). Even though it was not statistically significant, protein levels tended to increase during ischemia and reperfusion, indicating that the decrease in HIF1α during these time points was not caused by generalized protein degeneration in the samples.

### 3.3. RT-qPCR

**Figure 6:**
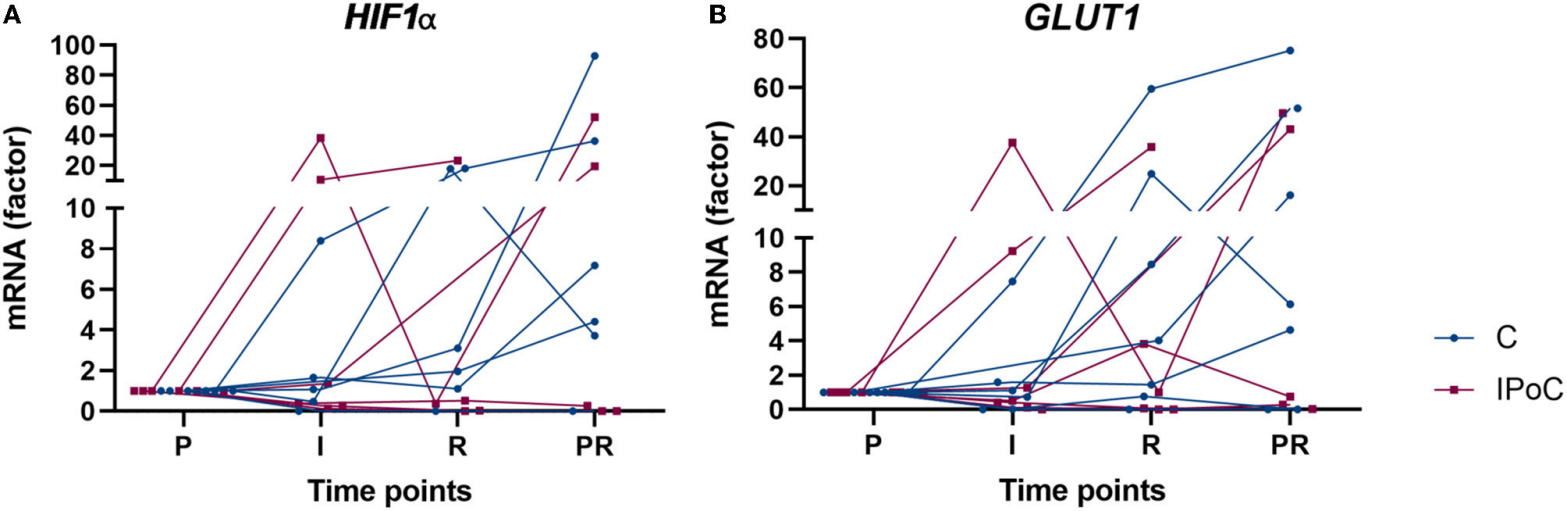
Individual value plots of qPCR results of HIF1α **(A)** and GLUT1 **(B)** expressed compared to pre-ischemia (P) during I, ischemia; R, reperfusion and in PR, proximal intestinal segment at reperfusion. In the control group, undelayed reperfusion was initiated following ischemia. In group IPoC, postconditioning was performed by clamping the mesenteric vessels after ischemia. No statistically significant differences could be detected between the time points and groups.

No significant differences could be found between the groups and time points for *EGLN1, EGLN3, GLUT1*, and *HIF1A* (Table 4). Although no statistical significance was detectable, the majority of horses showed an increase in *GLUT1* and *HIF1A* at the reperfusion time point in the post-ischemic and/or in the proximal sample (Figure 6).

**Table 4:** qPCR results relative to pre-ischemia.

	**Control**	**IPoC**
**EGLN1**		
Pre-ischemia	1	1
Ischemia	1.9 ± 3.1	1.8 ± 2.5
Reperfusion	0.5 ± 0.2	5.0 ± 11.9
Proximal	1.3 ± 1.1	5.1 ± 7.9
**EGLN3**		
Pre-ischemia	1	1
Ischemia	1.0 ± 1.0	1.4 ± 1.3
Reperfusion	2.6 ± 3.3	0.8 ± 0.8
Proximal	10.1 ± 17.3	24.3 ± 33.5
**HIF1**α		
Pre-ischemia	1	1
Ischemia	2.3 ± 3.5	8.5 ± 15.5
Reperfusion	7.0 ± 8.5	4.9 ± 10.3
Proximal	24.0 ± 36.1	14.3 ± 22.7
**GLUT1**		
Pre-ischemia	1	1
Ischemia	1.8 ± 2.8	7.0 ± 13.9
Reperfusion	14.2 ± 21.8	6.8 ± 14.3
Proximal	22.0 ± 29.6	18.7 ± 25.2

The table displays the mean ± standard deviation of the qPCR results displayed as factor relative to the pre-ischemia levels. There were no significant differences between the groups or time points.

## 4. Discussion

This study documented the immunoreactivity pattern of HIF1α and−2α in sequential measurements during experimental intestinal ischemia in the horse. The main findings were that the nuclear and cytoplasmic HIF1α immunoreactivity increased during reperfusion compared to pre-ischemia. Contrarily, the perinuclear HIF1α stained granules as well as the HIF1α protein levels determined with ELISA were decreased during reperfusion, and no significant upregulation of *HIF1A* and its target genes could be detected. The HIF2α distribution and immunoreactivity did not show any progression during ischemia or reperfusion. Therefore, we could only partially accept our hypothesis that HIF1α and HIF2α levels would increase during ischemia and reperfusion. There was no difference in HIF1α and−2α distribution between the groups, and the HIF1α protein expression was lower in the control group compared to the postconditioned group during both ischemia and reperfusion. With the latter difference already occurring prior to the implementation of IPoC, this difference cannot be related to this intervention. Consequently, we rejected the hypothesis that the HIF response would be more pronounced in the animals subjected to postconditioning.

Looking more closely at the HIF1α immunoreactivity pattern in the jejunum, some degree of staining was present in all cell types, as previously reported in the equine small intestine (18). The increased enterocyte immunoreactivity score over time correlates with a rodent study that reported increased numbers of positive cells in injured villi (33). The increased nuclear staining was found in the villi only, possibly explained by the higher level of hypoxia that is to be expected in the mucosal villi compared to the crypts, leading to increased stabilization and translocation to the nucleus (34, 35). Interestingly, the increased nuclear and cytoplasmic staining was not seen directly after ischemia, but after 2 h of reperfusion. Comparing this to the timing of HIF1α expression as reported in the literature, intestinal protein levels have been shown to be increased at 2–12 h following hypoxia (17, 26, 36). The histomorphological examination of these samples showed a significant degree of epithelial separation and hemorrhage during ischemia, that did not progress significantly during reperfusion (27). Relating these findings to the HIF1α immunoreactivity, this indicates that the first occurrence of histomorphological mucosal damage at ischemia does not necessarily correlate with an increase in enterocyte HIF1α immunoreactivity seen at reperfusion. Alternatively, non-hypoxic HIF stabilization needs to be taken into account, knowing that inflammatory cytokines such as IL-1 and tumor necrosis factor can increase HIF1α activity (37–39). Considering that these samples were shown to exhibit a major neutrophilic influx during reperfusion (40), this inflammatory response may have played a role in eliciting a pronounced HIF1α response at this time point. Furthermore, it has been shown that the exposure to bacteria may increase HIF1α protein expression or prevent the expected decrease in HIF1α protein levels during reoxygenation (15). Another consideration for the interpretation of the current results is the use of isoflurane for maintaining general anesthesia in these horses. This could influence the HIF levels, as volatile anesthetics may elicit protective effects against IRI through HIF signaling (41–44).

A remarkable finding was the presence of perinuclear hyperintense HIF staining in a varying proportion of villus and crypt enterocytes, which we referred to as perinuclear granules (Figure 1). The negative isotype controls did not exhibit any staining, suggesting that this pattern is not a form of unspecific binding. Furthermore, a granular HIF1α distribution in the cytoplasm has previously been reported in the colon (45) and liver (46). This phenomenon may represent the accumulation of HIF1α in cytosolic vesicles. Another study investigating HIF1α in cell cultures localized the protein in the sub-membranous compartment and within cytosolic vesicles. This was linked to a reduced activation of its downstream pathway, indicating a non-functional HIF1α variant in response to decreased cytoplasmic degradation (47). This concept could apply to the enterocytes in the current study, although this cannot be concluded without further investigation. This may be supported by the finding that the perinuclear granules were significantly decreased during the time that there was a significant increase in cytoplasmic immunoreactivity after reperfusion. This could be associated with increased cytoplasmic stabilization seen at this time-point, possibly indicating a shift away from a focal collection.

Contrarily to the increasing immunoreactivity seen over time in the histological examination, protein levels measured with the ELISA decreased in the control group during reperfusion (Figure 4). The histology score analyzed enterocyte staining only, whereas the quantitative tissue examination was performed on the complete stripped mucosa, which may in part explain the differences between these two analyses. Moreover, the intensely stained perinuclear granules may contribute to the protein content. These were decreased during reperfusion, possibly influencing the total protein concentration of the tissue sample. Another factor that could have influenced these results, is that the point of maximal HIF1α accumulation may have been missed within the 120 min of time between the ischemia and reperfusion sample, or that significant upregulation would have occurred later, beyond the last sampling time point (17, 26, 36). Furthermore, we cannot exclude the possibility that differences in tissue processing could have influenced the degradation of HIF1α. The histology samples were the first to be fixed in formalin, after which the tissue was processed for protein and RNA analysis by mucosa stripping and snap-freezing the samples. Even though the latter only took a few minutes longer, the cytoplasmic stabilization of HIF1α *in vivo* could have been reversed by exposure of the *ex vivo* samples to oxygen in the air. In such instance, HIF degradation could possibly be faster in samples with erosion of the epithelial border due to faster oxygen diffusion compared to samples with intact epithelium. It is unlikely that the decreased HIF1α levels in the samples can be attributed to generalized protein degradation, as the HSP-70 levels did not show such a decrease (Figure 5).

Regarding the transcript expression, no statistically significant differences could be detected (Table 4). This may be attributed to the timing of the samples, as increased mRNA levels have been found to reach the maximum during the ischemic phase (48). Furthermore, the regulation of HIF1α levels may predominantly be posttranscriptional (8). Therefore, the higher HIF levels seen in the histology samples during reperfusion may be the result of increased stabilization instead of upregulation, although no significant upregulation of the target genes *GLUT1, EGLN1*, and *3* was found. A comparable pattern was seen in another study investigating IRI in horses, with only partial and delayed upregulation of HIF-targets (49). On the other hand, the analysis of the complete mucosa instead of only the enterocytes could have caused smaller differences to go undetected. Furthermore, the high interindividual variability of the qPCR results combined with the small sample size may have prevented the detection of differences between the groups and time points.

The HIF2α immunoreactivity pattern demonstrated mainly cytoplasmic staining of the enterocytes (Figure 2). The majority of the stromal cells were negative, similar to the results of a rodent study (22). This study also reported weak and inconsistent staining in the jejunum compared to the duodenum. This comparison could not be made in the current study, as only jejunum samples were taken. Interestingly, no significant change in immunoreactivity could be noted over time. The only finding that seemed related to the time point was the incidental change in perinuclear staining in the villus enterocytes at epithelial erosion fronts. However, this was not consistently present, and it may also be related to the intracellular changes that occur at the time of epithelial separation, without a direct effect on HIF2α. These results suggest that HIF2α is not of significance in the hypoxia signaling in the current model of intestinal ischemia in horses. However, there are no other equine studies available, and comparable intestinal histological studies or studies in *in vivo* ischemia models are lacking, limiting further comparison of results.

Three of 14 horses exhibited a consistent and distinct intense perinuclear staining pattern in nearly all enterocytes throughout all samples, whereas other horses demonstrated perinuclear staining in only a small proportion of the enterocytes (Figure 3). Nearly all samples also demonstrated a granular HIF2α immunoreactivity pattern in the tunica muscularis. As discussed for the HIF1α results, this staining pattern may represent the assembly in vesicles. The fact that all samples of these individuals showed the same distribution, independent of timing or ischemia, suggests an association with the individual horses and not with the experimental model. It has been shown that HIF2α expression can be related to age, with lower levels in aged gingival tissues (50). However, looking at age, breed and sex of these three horses, no explanation could be found for the different HIF2α distribution pattern. The horses did not differ from the other animals in degree of mucosal injury or inflammation. Furthermore, there were no disparities in test group, timing of the experiment, involved personnel or sample processing. Therefore, the cause this for phenomenon remains unresolved, and requires further investigation.

A direct comparison between the HIF1α and−2α scores was not performed, because the difference in antibody type (monoclonal vs. polyclonal) and dilution would preclude a reliable comparison of staining intensity. Nevertheless, looking at the immunoreactivity patterns of both factors, clear differences could be identified. Firstly, nuclear immunoreactivity for HIF1α was seen in all cell types, while there was hardly any nuclear staining for HIF2α. Furthermore, HIF1α showed more intense staining in the crypts compared to the villi, yet for HIF2α this was the other way around. Moreover, the HIF2α stained slides exhibited a higher proportion of enterocytes with focal perinuclear staining, and the focal granular staining in the tunica muscularis was only found in these slides.

Previous studies have found evidence for upregulation of HIF1α being of significance for the protective action of ischemic preconditioning (IPC) and IPoC in laboratory animals (26, 29, 51). HIF1α was shown to be higher after both intestinal and cardiac IPoC, with subsequent upregulation of microRNA-21 as mediator (26, 29). Contrarily, another study investigating IPoC in myocardial ischemia showed that microRNA-214 may participate in the protective function of IPoC by down-regulating HIF1α (30). These conflicting results indicate that the association between HIF1α and the protective action of ischemic conditioning is not set in stone. In the current study, we could not find a significant difference between the control group and the group undergoing postconditioning in HIF1α and−2α immunoreactivity pattern in the intestine. Regarding the mucosal HIF1α protein levels, the control group had lower levels during ischemia and reperfusion. Because the groups were identically treated until the initiation of reperfusion, the group difference at ischemia cannot be attributed to an effect of IPoC. Therefore, these results should be interpreted with caution, and it cannot lead to the conclusion that IPoC results in higher HIF1α protein levels. In our previous study, we found some indicators for a protective effect of IPoC, yet the parameters for oxidative stress and inflammation did not differ between the groups (27, 40). This could possibly account for the absence of a significant effect on HIF1α, considering the relationship between the HIF1α response, oxidative stress, and inflammation (35, 37–39, 52).

The main limitations of this study are the small sample size and the variability between the horses. This in combination with the multiple complex pathways of HIF activation and possible HIF degradation in samples, complicates the interpretation of results. The semi-quantitative analysis of the immunohistology may limit the detection of smaller differences and may be prone to subjectivity. Nevertheless, the slides were reviewed in a blinded manner, and an observer bias would be present in all samples. Furthermore, reference microscopic images were used to standardize this evaluation. For the semi-quantitative score, it was chosen to evaluate the enterocytes alone and not the complete mucosa, as the other cell types hardly showed any change over time, which would decrease the sensitivity for detecting changes in the enterocytes. A more objective method of detection of color differences with commercially available software was evaluated, yet this precluded the differentiation between the cell types, and therefore the manual semi-quantitative scoring was preferred. A semi-quantitative score of the tunica muscularis was also included because differences appeared to be present during the subjective evaluation of the slides, and hypoxia or inflammation related changes in this intestinal section may be related to the occurrence of postoperative ileus. However, there were no significant changes between the different phases of ischemia. Compared to clinical cases with strangulating intestinal disease, the experimental trial has a relatively short time span with concurrent lower grades of intestinal IRI. Consequently, a HIF response that may be significant in clinical colic cases could go undetected in the current experimental set up. Another limitation of the study is the lack of a quantitative analysis for HIF2α, which was decided against because the immunohistological evaluation showed no progression or change whatsoever.

In conclusion, the enterocyte changes in HIF1α immunoreactivity over time indicate that this transcription factor may play a role in the intestinal response to ischemia in horses. The protein expression did not mirror these findings, possibly due to inclusion of more cell types or differences in sample preparation. HIF2α did not show any progression during ischemia or reperfusion and no nuclear staining was observed, suggesting that this transcription factor does not modulate the effect of hypoxia in small intestinal ischemia in this species. A distinct perinuclear focal HIF2α staining pattern was associated with individual horses and not with time points, requiring further investigation to determine its significance. We could not detect a difference in HIF1α and−2α immunoreactivity between the treatment groups. This may indicate that these factors are not relevant for postconditioning in the current experimental model, although these results need to be interpreted with care due to the high variability in some of the tested variables. Investigating more downstream targets and the use of organoids as controlled environment in future studies could help clarify the exact role of HIF expression and distribution in the equine intestine.

## Data availability statement

The datasets presented in this study can be found in online repositories. The names of the repository/repositories and accession number(s) can be found at: https://doi.org/10.17632/mxhhxpvpvj.2, Mendeley Data.

## Ethics statement

The animal study was reviewed and approved by Ethics Committee for Animal Experiments of Lower Saxony, Germany.

## Author contributions

Study design: NV, NB, MK-B, MH-T, and SK. Study execution: NV, NB, KD, MH-T, FD, and SK. Data analysis: NV, NB, and FD. Data interpretation: NV, NB, MK-B, KD, MH-T, CP, FD, and SK. Preparation of the manuscript and figures: NV. All authors read and approved the final manuscript.

## References

[1] Tinker MK White N Lessard P Thatcher C Pelzer K Davis B. Prospective study of equine colic incidence and mortality. Equine Vet J. (1997) 29:448–53. 10.1111/j.2042-3306.1997.tb03157.x 9413717

[2] Tirpe AA Gulei D Ciortea SM Crivii C Berindan-Neagoe I. Hypoxia: overview on hypoxia-mediated mechanisms with a focus on the role of HIF genes. Int J Mol Sci. (2019) 20:6140. 10.3390/ijms20246140 31817513 PMC6941045

[3] Semenza GL. Regulation of mammalian O_2_ homeostasis by hypoxia-inducible factor 1. Annu Rev Cell Dev Biol. (1999) 15:551–78. 10.1146/annurev.cellbio.15.1.551 10611972

[4] Chan DA Sutphin PD Yen SE Giaccia AJ. Coordinate regulation of the oxygen-dependent degradation domains of hypoxia-inducible factor 1 alpha. Mol Cell Biol. (2005) 25:6415–26. 10.1128/MCB.25.15.6415-6426.2005 16024780 PMC1190339

[5] Wang GL Jiang BH Rue EA Semenza GL. Hypoxia-inducible factor 1 is a basic-helix-loop-helix-PAS heterodimer regulated by cellular O_2_ tension. Proc Natl Acad Sci U S A. (1995) 92:5510–4. 10.1073/pnas.92.12.5510 7539918 PMC41725

[6] Singhal R Shah YM. Oxygen battle in the gut: hypoxia and hypoxia-inducible factors in metabolic and inflammatory responses in the intestine. J Biol Chem. (2020) 295:10493–505. 10.1074/jbc.REV120.011188 32503843 PMC7383395

[7] Jing X Yang F Shao C Wei K Xie M Shen H. Role of hypoxia in cancer therapy by regulating the tumor microenvironment. Mol Cancer. (2019) 18:157. 10.1186/s12943-019-1089-9 31711497 PMC6844052

[8] Akhtar M Sutherland A Huang H Ploeg R Pugh C. The role of hypoxia-inducible factors in organ donation and transplantation: the current perspective and future opportunities. Am J Transplant. (2014) 14:1481–7. 10.1111/ajt.12737 24909061

[9] Saeedi BJ Kao DJ Kitzenberg DA Dobrinskikh E Schwisow KD Masterson JC. HIF-dependent regulation of claudin-1 is central to intestinal epithelial tight junction integrity. Mol Biol Cell. (2015) 26:2252–62. 10.1091/mbc.E14-07-1194 25904334 PMC4462943

[10] Synnestvedt K Furuta GT Comerford KM Louis N Karhausen J Eltzschig HK. Ecto-5'-nucleotidase (CD73) regulation by hypoxia-inducible factor-1 mediates permeability changes in intestinal epithelia. J Clin Invest. (2002) 110:993–1002. 10.1172/JCI0215337 12370277 PMC151145

[11] Yang S Yu M Sun L Xiao W Yang X Sun L. Interferon-γ-induced intestinal epithelial barrier dysfunction by NF-κB/HIF-1α pathway. J Interferon Cytokine Res. (2014) 34:195–203. 10.1089/jir.2013.0044 24237301

[12] Bäcker V Cheung F-Y Siveke JT Fandrey J Winning S. Knockdown of myeloid cell hypoxia-inducible factor-1α ameliorates the acute pathology in DSS-induced colitis. PLoS ONE. (2017) 12:e0190074. 10.1371/journal.pone.0190074 29261815 PMC5738114

[13] Karhausen J Furuta GT Tomaszewski JE Johnson RS Colgan SP Haase VH. Epithelial hypoxia-inducible factor-1 is protective in murine experimental colitis. J Clin Invest. (2004) 114:1098–106. 10.1172/JCI200421086 15489957 PMC522241

[14] Grenz A Clambey E Eltzschig HK. Hypoxia signaling during intestinal ischemia and inflammation. Curr Opin Crit Care. (2012) 18:178–85. 10.1097/MCC.0b013e3283514bd0 22322265 PMC3855266

[15] Koury J Deitch EA Homma H Abungu B Gangurde P Condon MR. Persistent HIF-1α activation in gut ischemia/reperfusion injury: potential role of bacteria and lipopolysaccharide. Shock. (2004) 22:270–7. 10.1097/01.shk.0000135256.67441.3f 15316398

[16] Kannan KB Colorado I Reino D Palange D Lu Q Qin X. Hypoxia-inducible factor plays a gut-injurious role in intestinal ischemia reperfusion injury. Am J Physiol Gastrointest Liver Physiol. (2011) 300:G853–61. 10.1152/ajpgi.00459.2010 21183660 PMC3094138

[17] Feinman R Deitch EA Watkins AC Abungu B Colorado I Kannan KB. HIF-1 mediates pathogenic inflammatory responses to intestinal ischemia-reperfusion injury. Am J Physiol Gastrointest Liver Physiol. (2010) 299:G833–43. 10.1152/ajpgi.00065.2010 20689059 PMC2957330

[18] Bauck AG Grosche A Morton AS Vickroy TW Freeman DE. Effect of lidocaine on in ammation in equine jejunum subjected to manipulation only and remote to intestinal segments subjected to ischemia. Am J Vet Res. (2017) 78:977–89. 10.2460/ajvr.78.8.977 28738006

[19] De Ceulaer K Delesalle C Van Elzen R Van Brantegem L Weyns A Van Ginneken C. Morphological data indicate a stress response at the oral border of strangulated small intestine in horses. Res Vet Sci. (2011) 91:294–300. 10.1016/j.rvsc.2010.11.020 21216416

[20] Mastrogiannaki M Matak P Keith B Simon MC Vaulont S Peyssonnaux C. HIF-2α, but not HIF-1α, promotes iron absorption in mice. J Clin Invest. (2009) 119:1159–66. 10.1172/JCI38499 19352007 PMC2673882

[21] Giatromanolaki A Sivridis E Maltezos E Papazoglou D Simopoulos C Gatter KC. Hypoxia inducible factor 1alpha and 2alpha overexpression in inflammatory bowel disease. J Clin Pathol. (2003) 56:209–13. 10.1136/jcp.56.3.209 12610101 PMC1769899

[22] Wiesener MS Jürgensen JS Rosenberger C Scholze C Hörstrup JH Warnecke C. Widespread, hypoxia-inducible expression of HIF-2α in distinct cell populations of different organs. FASEB J. (2003) 17:271–3. 10.1096/fj.02-0445fje 12490539

[23] Makino Y Cao R Svensson K Bertilsson G Asman M Tanaka H. Inhibitory PAS domain protein is a negative regulator of hypoxia-inducible gene expression. Nature. (2001) 414:550–4. 10.1038/35107085 11734856

[24] Duan C. Hypoxia-inducible factor 3 biology: complexities and emerging themes. Am J Physiol Cell Physiol. (2016) 310:C260–9. 10.1152/ajpcell.00315.2015 26561641

[25] Zhao ZQ Corvera JS Halkos ME Kerendi F Wang NP Guyton RA. Inhibition of myocardial injury by ischemic postconditioning during reperfusion: comparison with ischemic preconditioning. Am J Physiol Heart Circ Physiol. (2003) 285:579–88. 10.1152/ajpheart.01064.2002 12860564

[26] Jia Z Lian W Shi H Cao C Han S Wang K. Ischemic postconditioning protects against intestinal ischemia/reperfusion injury *via* the HIF-1alpha/miR-21 axis. Sci Rep. (2017) 7:16190. 10.1038/s41598-017-16366-6 29170412 PMC5700993

[27] Verhaar N Breves G Hewicker-Trautwein M Pfarrer C Rohn K Burmester M. The effect of ischaemic postconditioning on mucosal integrity and function in equine jejunal ischaemia. Equine Vet J. (2022) 54:427–37. 10.1111/evj.13450 34003501

[28] Zhao H-X Wang X-L Wang Y-H Wu Y Li X-Y Lv X-P. Attenuation of myocardial injury by postconditioning: role of hypoxia inducible factor-1α. Basic Res Cardiol. (2010) 105:109. 10.1007/s00395-009-0044-0 19597757

[29] Liu Y Nie H Zhang K Ma D Yang G Zheng Z. A feedback regulatory loop between HIF-1α and miR-21 in response to hypoxia in cardiomyocytes. FEBS Lett. (2014) 588:3137–46. 10.1016/j.febslet.2014.05.067 24983504

[30] Wan D Zhang Z Yang H. Cardioprotective effect of miR-214 in myocardial ischemic postconditioning by down-regulation of hypoxia inducible factor 1, alpha subunit inhibitor. Cell Mol Biol. (2015) 61:1. 26025394

[31] Bradford MM. A rapid and sensitive method for the quantitation of microgram quantities of protein utilizing the principle of protein-dye binding. Anal Biochem. (1976) 72:248–54. 10.1016/0003-2697(76)90527-3 942051

[32] Pfaffl MW Horgan GW Dempfle L. Relative expression software tool (REST) for group-wise comparison and statistical analysis of relative expression results in real-time PCR. Nucleic Acids Res. (2002) 30:e36. 10.1093/nar/30.9.e36 11972351 PMC113859

[33] Tuboly E Futakuchi M Varga G Érces D Tokés T Mészáros A. C5a inhibitor protects against ischemia/reperfusion injury in rat small intestine. Microbiol Immunol. (2016) 60:35–46. 10.1111/1348-0421.12338 26576826 PMC4819679

[34] Blikslager AT. The Equine Acute Abdomen. Hoboken, NJ: John Wiley and Sons. (2017). 10.1002/9781119063254

[35] Krock BL Skuli N Simon MC. Hypoxia-induced angiogenesis: good and evil. Genes Cancer. (2011) 2:1117–33. 10.1177/1947601911423654 22866203 PMC3411127

[36] Ji Z-P Li Y-X Shi B-X Zhuang Z-N Yang J-Y Guo S. preconditioning protects Ca2+-ATPase activation of intestinal mucosal cells against R/I injury in a rat liver transplantation model. World J Gastroenterol. (2018) 24:360. 10.3748/wjg.v24.i3.360 29391758 PMC5776397

[37] Scharte M Han X Bertges DJ Fink MP Delude RL. Cytokines induce HIF-1 DNA binding and the expression of HIF-1-dependent genes in cultured rat enterocytes. Am J Physiol Gastrointest Liver Physiol. (2003) 284:G373–84. 10.1152/ajpgi.00076.2002 12388200

[38] Garcia-Vasquez C Fernandez-Acenero MJ Garcia Gomez-Heras S Pastor C. Fibrin patch influences the expression of hypoxia-inducible factor-1alpha and nuclear factor-kappaBp65 factors on ischemic intestinal anastomosis. Exp Biol Med. (2018) 243:803–8. 10.1177/1535370218777216 29932372 PMC6022912

[39] Hellwig-Bürgel T Rutkowski K Metzen E Fandrey J Jelkmann W. Interleukin-1β and tumor necrosis factor-α stimulate DNA binding of hypoxia-inducible factor-1. Blood. (1999) 94:1561–7. 10.1182/blood.V94.5.1561.417a06_1561_1567 10477681

[40] Verhaar N de Buhr N von Köckritz-Blickwede M Hewicker-Trautwein M Pfarrer C Mazzuoli-Weber G. Ischaemic postconditioning reduces apoptosis in experimental jejunal ischaemia in horses. BMC Vet Res. (2021) 17:1–14. 10.1186/s12917-021-02877-y 33902575 PMC8077964

[41] Ye Z Guo Q Xia P Wang N Wang E Yuan Y. Sevoflurane postconditioning involves an up-regulation of HIF-1α and HO-1 expression *via* PI3K/Akt pathway in a rat model of focal cerebral ischemia. Brain Res. (2012) 1463:63–74. 10.1016/j.brainres.2012.04.050 22580326

[42] Raphael J Zuo Z Abedat S Beeri R Gozal Y. Isoflurane preconditioning decreases myocardial infarction in rabbits *via* up-regulation of hypoxia inducible factor 1 that is mediated by mammalian target of rapamycin. J Am Soc Anesthesiol. (2008) 108:415–25. 10.1097/ALN.0b013e318164cab1 18292679

[43] Wang C Weihrauch D Schwabe DA Bienengraeber M Warltier DC Kersten JR. Extracellular signal-regulated kinases trigger isoflurane preconditioning concomitant with upregulation of hypoxia-inducible factor-1α and vascular endothelial growth factor expression in rats. Anesth Analg. (2006) 103:281–8. 10.1213/01.ane.0000226094.94877.98 16861403

[44] Ngamsri K-C Fabian F Fuhr A Gamper-Tsigaras J Straub A Fecher D. Sevoflurane exerts protective effects in murine peritonitis-induced sepsis *via* hypoxia-inducible factor 1α/adenosine A2B receptor signaling. Anesthesiology. (2021) 135:136–50. 10.1097/ALN.0000000000003788 33914856

[45] Mariani F Sena P Marzona L Riccio M Fano R Manni P. Cyclooxygenase-2 and hypoxia-inducible factor-1α protein expression is related to inflammation, and up-regulated since the early steps of colorectal carcinogenesis. Cancer Lett. (2009) 279:221–9. 10.1016/j.canlet.2009.02.001 19268443

[46] Li S Yao D Wang L Wu W Qiu L Yao M. characteristics of hypoxia-inducible factor-1α and its clinical values in diagnosis and prognosis of hepatocellular carcinoma. Hepat Mon. (2011) 11:821–8. 10.5812/kowsar.1735143X.1305 22224081 PMC3234574

[47] Armando F Gambini M Corradi A Giudice C Pfankuche VM Brogden G. Oxidative stress in canine histiocytic sarcoma cells induced by an infection with canine distemper virus led to a dysregulation of HIF-1α downstream pathway resulting in a reduced expression of VEGF-B *in vitro*. Viruses. (2020) 12:200. 10.3390/v12020200 32054075 PMC7077254

[48] Nishie H Takahashi T Inoue K Shimizu H Morimatsu H Toda Y. Site-specific induction of intestinal hypoxia-inducible factor-1α after hemorrhagic shock. Mol Med Rep. (2009) 2:149–52. 10.3892/mmr_00000075 21475804

[49] Dengler F Sternberg F Grages M Kästner SB Verhaar N. Adaptive mechanisms in no flow vs. low flow ischemia in equine jejunum epithelium: different paths to the same destination. Front Vet Sci. 9:947482. (2022). 10.3389/fvets.2022.947482 36157182 PMC9493374

[50] Ebersole JL Novak MJ Orraca L Martinez-Gonzalez J Kirakodu S Chen KC. Hypoxia-inducible transcription factors, HIF1A and HIF2A, increase in aging mucosal tissues. Immunology. (2018) 154:452–64. 10.1111/imm.12894 29338076 PMC6002220

[51] Chen Y Lee S-H Tsai Y-H Tseng S-H. Ischemic preconditioning increased the intestinal stem cell activities in the intestinal crypts in mice. J Surg Res. (2014) 187:85–93. 10.1016/j.jss.2013.10.001 24176207

[52] Movafagh S Crook S Vo K. Regulation of hypoxia-inducible factor-1a by reactive oxygen species: new developments in an old debate. J Cell Biochem. (2015) 116:696–703. 10.1002/jcb.25074 25546605

