# Supplementary material for: Hypoxia signaling in the equine small intestine: Expression and distribution of hypoxia inducible factors during experimental ischemia
Source: Front Vet Sci. 2023 Feb 24;10:1110019. doi: 10.3389/fvets.2023.1110019 (PMC9998946; doi:10.3389/fvets.2023.1110019)
Supplement: Supplementary file 1 [file Data_Sheet_1.PDF]

Supplementary Item 1

Hypoxia Inducible Factor 1α and -2α Immunohistochemistry

Hypoxia Inducible Factor 1α

The following light microscopic images depict the positive and equine jejunal negative controls of immunohistochemical staining for Hypoxia Inducible Factor 1α with polyclonal rabbit antibodies, as well as representative images for the following semi-quantitative immunoreactivity score for cytoplasmic and nuclear staining of the jejunal enterocytes: grade 0 – no staining; 1 – weak staining (hardly visible); 2 – mild staining (light brown); 3 – moderate staining (middle brown, lighter than the hyperintense neutrophils and endothelial cells); 4 – intense staining (dark brown, comparable to hyperintense neutrophils and endothelial cells). The scale bar indicates 50 µm.

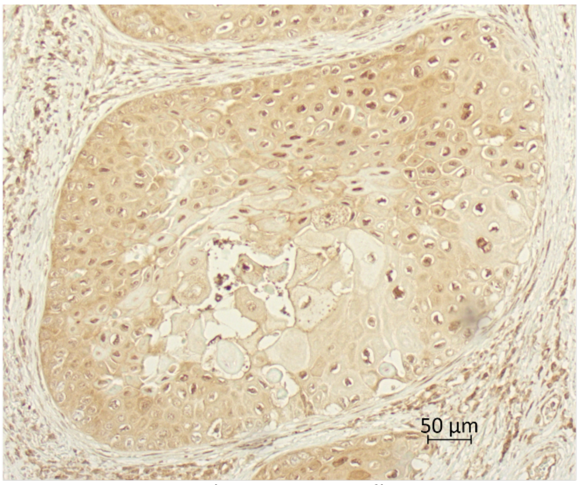

Positive control – squamous cell carcinioma
(equine preputial tissue)

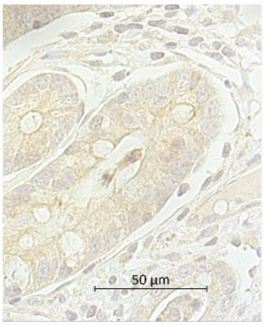

Negative control - Rabbit IgG

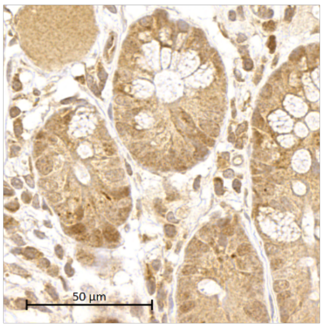

Cytoplasm 1 / Nucleus 1

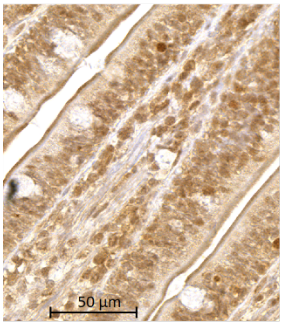

Cytoplasm 1 / Nucleus 2

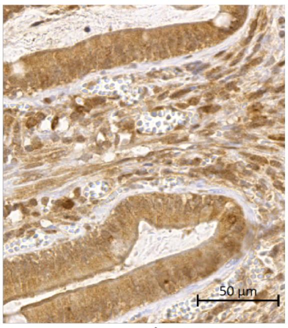

Cytoplasm 2 / Nucleus 2

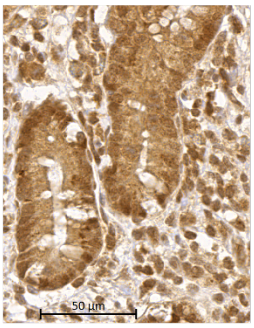

Cytoplasm 2 / Nucleus 3

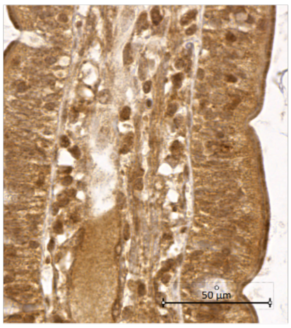

Cytoplasm 3 / Nucleus 3

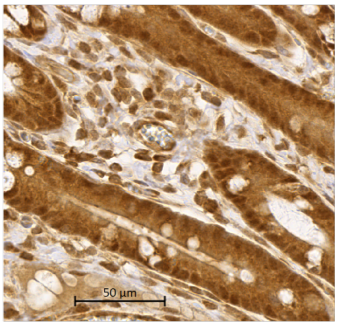

Cytoplasm 3 / Nucleus 4

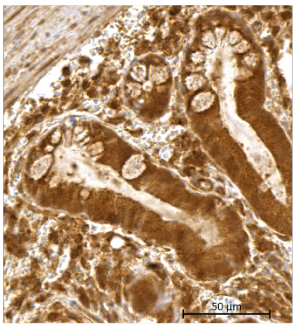

Cytoplasm 4 / Nucleus 4

Hypoxia Inducible Factor 2α
The following light microscopic images depict the positive and equine jejunal negative controls of immunohistochemical staining for Hypoxia Inducible Factor 2α with monoclonal mouse antibodies, as well as representative images for the following semi-quantitative immunoreactivity score for staining of the cytoplasm of the jejunal enterocytes: grade 0 – no staining; 1 – weak staining (hardly visible); 2 – mild staining (light brown); 3 – moderate staining (middle brown). There was no hyperintense or nuclear staining in any of the enterocytes. The scale bar indicates 50 µm.

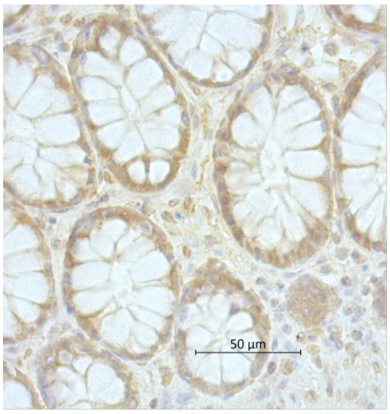

Positive control – human colon

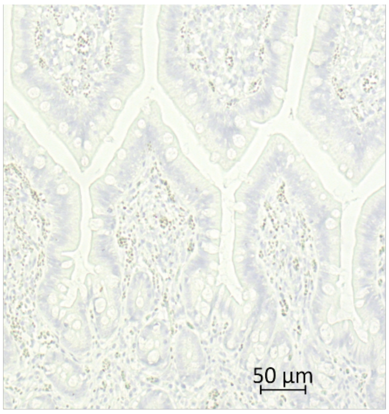

Negative control – Mouse IgG1

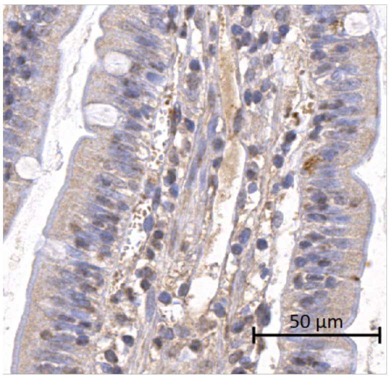

Cytoplasm 1 / Nucleus 0

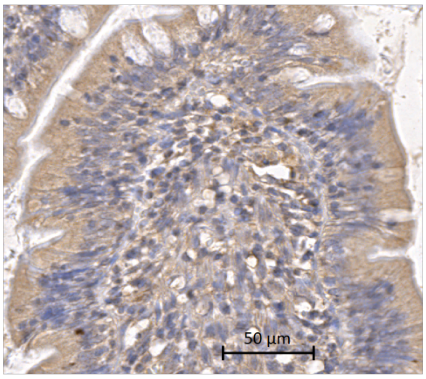

Cytoplasm 2 / Nucleus 0

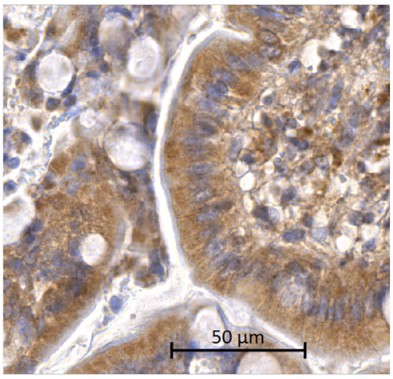

Cytoplasm 3 / Nucleus 0
