# Supplementary material for: Hypoxia signaling in the equine small intestine: Expression and distribution of hypoxia inducible factors during experimental ischemia
Source: Front Vet Sci. 2023 Feb 24;10:1110019. doi: 10.3389/fvets.2023.1110019 (PMC9998946; doi:10.3389/fvets.2023.1110019)
Supplement: Supplementary file 2 [file Data_Sheet_2.PDF]

## Supplementary Item 2

Realtime PCR - threshold cycle

| | RPL4 | | HPRT1 | | EGLN1 | | EGLN3 | | GLUT1 | | HIF1A | |
|---|---|---|---|---|---|---|---|---|---|---|---|---|
| | **Control** | | | | | | | | | | | |
| | **Ct mean** | **SD** | **Ct mean** | **SD** | **Ct mean** | **SD** | **Ct mean** | **SD** | **Ct mean** | **SD** | **Ct mean** | **SD** |
| **P** | **18.91** | 1.51 | **25.58** | 2.77 | **25.79** | 1.82 | **24.19** | 2.09 | **26.25** | 1.93 | **25.10** | 1.94 |
| **I** | **19.34** | 3.74 | **26.72** | 3.80 | **26.16** | 2.84 | **23.37** | 0.60 | **26.42** | 2.78 | **25.52** | 2.35 |
| **R** | **18.55** | 1.55 | **26.49** | 3.29 | **26.81** | 1.75 | **23.80** | 1.51 | **24.88** | 3.39 | **24.94** | 3.40 |
| **PR** | **19.06** | 1.06 | **26.61** | 2.79 | **26.16** | 2.01 | **25.47** | 5.47 | **25.01** | 2.76 | **24.91** | 2.34 |
| | **IPoC** | | | | | | | | | | | |
| **P** | **20.51** | 2.98 | **28.39** | 3.28 | **27.36** | 2.36 | **22.60** | 1.55 | **27.02** | 1.94 | **25.67** | 1.68 |
| **I** | **19.84** | 2.49 | **28.89** | 4.40 | **27.88** | 3.11 | **21.88** | 0.33 | **27.63** | 3.02 | **26.81** | 2.70 |
| **R** | **20.19** | 3.44 | **28.26** | 4.32 | **28.82** | 4.00 | **22.68** | 1.11 | **27.54** | 4.63 | **27.27** | 3.50 |
| **PR** | **22.18** | 2.95 | **29.63** | 3.02 | **28.42** | 2.11 | **23.63** | 1.69 | **27.26** | 2.93 | **27.41** | 3.73 |

P pre-ischemia; I Ischemia; R Reperfusion; PR proximal sample; $C_t$ threshold cycle; SD standard deviation
